# Oxidation of Uroporphyrinogens During Heme Synthesis: Role of Iron, Susceptibility and Consequences

**DOI:** 10.3390/biom16081188

**Published:** 2026-08-14

**Authors:** Andrew G. Smith

**Affiliations:** 1 Division of Genetics and Genome Biology, University of Leicester, Leicester LE1 7RH, UK; ags5@le.ac.uk or ags56@mrc-tox.cam.ac.uk; 2 Medical Research Council Toxicology Unit, University of Cambridge, Cambridge CB2 1TN, UK

**Keywords:** uroporphyrinogens, idiopathic oxidation, iron, susceptibility, porphyria

## Abstract

In the biosynthesis of heme the tetrapyrrole hydroxymethylbilane is converted enzymatically to uroporphyrinogen III whereas conversion to uroporphyrinogen I occurs spontaneously. Both are substrates for uroporphyrinogen decarboxylase (UROD) but only the III isomer is a precursor of heme. These porphyrinogens are easily oxidised to the respective uroporphyrins and trace amounts occur in the urine of healthy humans and animals. Large quantities of uroporphyrins I and III, as well as other oxidation products, occur in the liver and urine of patients with some porphyrias and after poisoning of people and animals by chemicals, such as hexachlorobenzene (HCB) and 2,3,7,8-tetrachorodibenzo-*p*-dioxin (TCDD). In the acquired disorder sporadic porphyria cutanea tarda (sPCT) and chemical-induced porphyria, hepatic UROD is inhibited, ostensibly by a partially oxidised uroporphyrinogen. The processes leading to oxidation of the uroporphyrinogens are interactions of a variety of external, internal and genetic factors. In some experimental systems, cytochrome P450 1A2 is implicated in the oxidation of uroporphyrinogens and uroporphyria and many patients and in vivo studies demonstrate the influence of iron. The article reviews the present state of knowledge of uroporphyrinogen oxidation, susceptibility and outcomes, and illustrates areas that require further exploration to explain fully the mechanisms of sPCT and the related uroporphyria caused by chemicals of toxic concern.

## 1. Introduction

The biosynthetic pathway of heme or related molecules is vital for most members of the biosphere [1]. Heme is a co-ordination complex of iron as a tetradentate ligand with the tetrapyrrole protoporphyrin IX formed from simple precursors via the first macrocyclic intermediate, uroporphyrinogen III. Pro- and eukaryotes that cannot synthesise heme themselves obtain it from other organisms or their surroundings [2]. Haemoproteins have many functions in all cells but these vary considerably depending on the exact intra molecular environment of the heme moiety, and include haemoglobin and cytochromes. Although in mammals the major proportion of heme synthesised is for haemoglobin, the liver contains approximately 15% of the body total heme required for mitochondrial cytochromes and haemoproteins in the metabolism of many bile acid and steroids and ingested compounds, including plant and synthetic chemicals. In plants the pathway is also used for the production of precursors of chlorophyll, ultimately incorporating Mg rather than Fe, and in bacteria to produce cobalamin.

**Figure 1:**
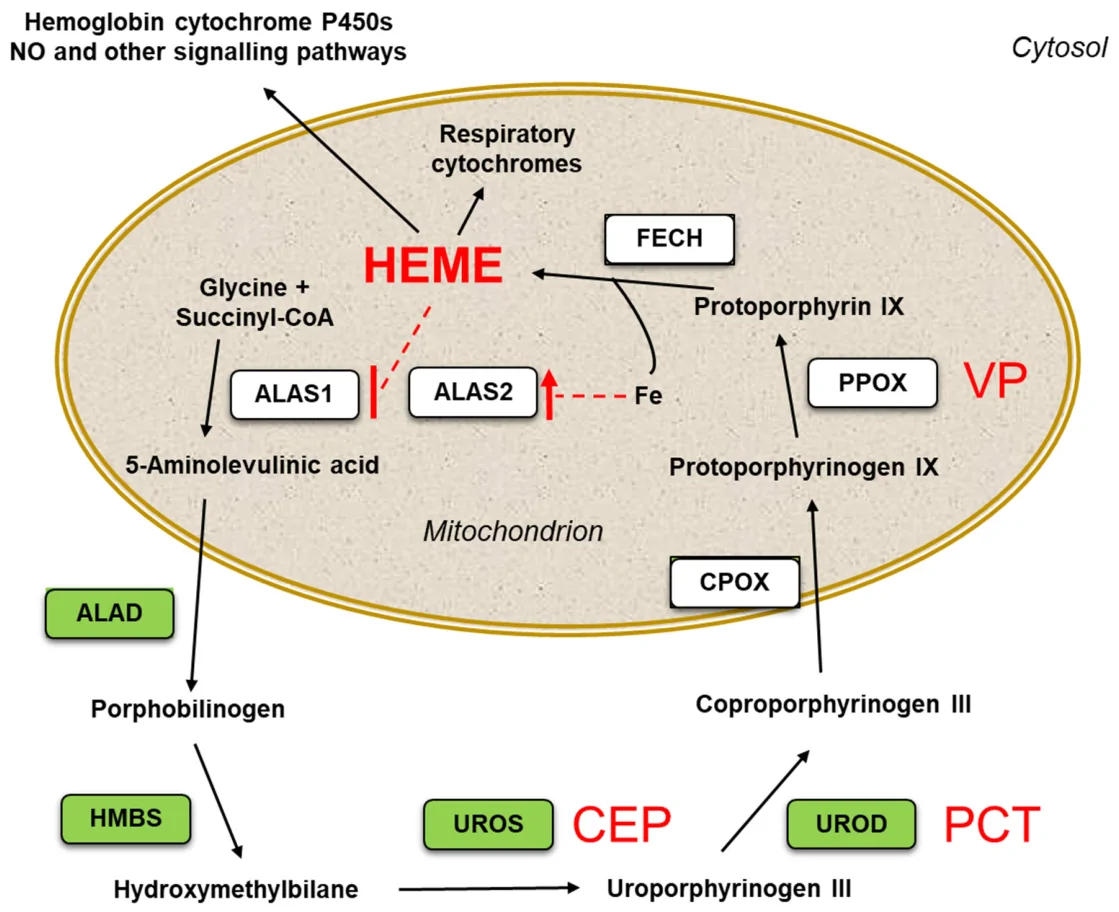
Heme synthesis in mammals. ALAS1, ‘house keeping’, 5-aminolevulinate synthase; ALAS2, erythropoietic 5-aminolevulinate synthase; ALAD, 5-aminolevulinate dehydratase; HMBS, 1-hydroxmethylbilane synthase. UROS, uroporphyrinogen III synthase; UROD, uroporphyrinogen decarboxylase; CPOX, coproporphyrinogen III oxidase; PPOX, protoporphyrinogen IX oxidase; FECH, ferrochelatase. *ALAS1* expression is considered to be modulated by a ‘regulatory heme’ pool, whereas *ALAS2* by availability of iron for erythropoiesis. Pathological mutations of the *PPOX* gene (variegate porphyria, VP) causes cutaneous deposition of protoporphyrin IX with extreme photosensitivity. Mutations or other factors leading to depressed activities of the enzymes UROS and UROD in humans cause disorders in which massive amounts of uroporphyrins accumulate in tissues especially skin or liver or both, as well as urinary excretion. CEP (mutation of *UROS* gene causing congenital erythropoietic porphyria). In porphyria cutanea tarda (PCT) UROD insufficiency can be due to nongenetic factors (sPCT) or in a minority a contribution from inherited mutations (fPCT). See Dickey et al. for details of other inherited human porphyrias [8]. Clinical disease and diagnoses can be complex due to the interaction of many biochemical and physiological factors besides inherited mutations.

In all organisms the complex structure of protoporphyrin IX is generated from a simple building block 5-aminolevulinic acid (5-ALA) in a series of eight stereospecific polymerisation and modification steps between mitochondria and cytosol (Figure 1). Formation of 5-ALA from the condensation of glycine with succinyl-CoA catalysed by 5-ALA synthase (5-ALAS) in the mitochondrion is a link with wider aspects of intermediary and energy metabolism [3,4,5,6]. Distinct genes for 5-ALAS are responsible for ‘house-keeping’ heme formation (*ALAS1*) and erythroid red blood cell production (*ALAS2*) allowing different control mechanisms. In plants and some other organisms 5-ALA is formed from glutamic acid. After export to the cytosol two molecules of 5-ALA yields a pyrrole product, porphobilinogen and head to tail addition of three more porphobilinogen units creates a linear tetrapyrrole, hydroxymethylbilane (HMB). Enzymatic cyclisation to an octacarboxylic porphyrinogen forms uroporphyrinogen III [7]. Decarboxylations of uroporphyrinogen III yields coproporphyrinogen III, which is imported back into the mitochondrion. Nonenzymatic cyclisation of hyroxymethylbilane also occurs but the product, uroporphyrinogen I, is not a precursor of heme. Oxidative decarboxylation of coproporphyrinogen III forms the divinyl molecule protoporphyrinogen IX. Oxidation to protoporphyrin IX occurs before Fe^2+^ is inserted to yield the final product heme.

Chemically, and in vivo, many of these intermediates are prone to oxidation especially in the presence of light but even in the dark [9]. In particular, the porphyrinogens are oxidised to their porphyrin analogues. The process and products of oxidation in vivo can have detectable clinical outcomes and in some circumstances the pathological consequences are severe. This occurs in some genetic and sporadic disorders of heme synthesis in humans and other mammals (the porphyrias) in which the intermediates and oxidation products accumulate in tissues especially skin and liver (Figure 1). Much is known about restricting the premature chemical oxidation of endogenous protoporphyrinogen IX before its intended conversion to protoporphyrin IX, that is likely in the reactive oxygen species (ROS) environment of the mitochondrion [10]. Under normal conditions oxidation of protoporphyrinogen IX to protoporphyrin IX in the mitochondrion is carefully controlled by protoporphyrinogen oxidase (PPOX) (Figure 1). In a genetic disorder with a *PPOX* mutation (variegate porphyria), protoporphyrin IX accumulates due to chemical oxidation of protoporphyinogen leading to severe hepatic injury and extreme cutaneous photosensitivity [11]. A similar phenomenon in deciduous plants was exploited commercially to develop a class of herbicides [12]. A similar outcome occurs in erythropoietic protoporphyria in which pathological variants of ferrochelatase are inherited [8]. Enhancement of protoporphyrin IX levels in targeted tissues is sometimes used in photodynamic therapy [13].

**Figure 2:**
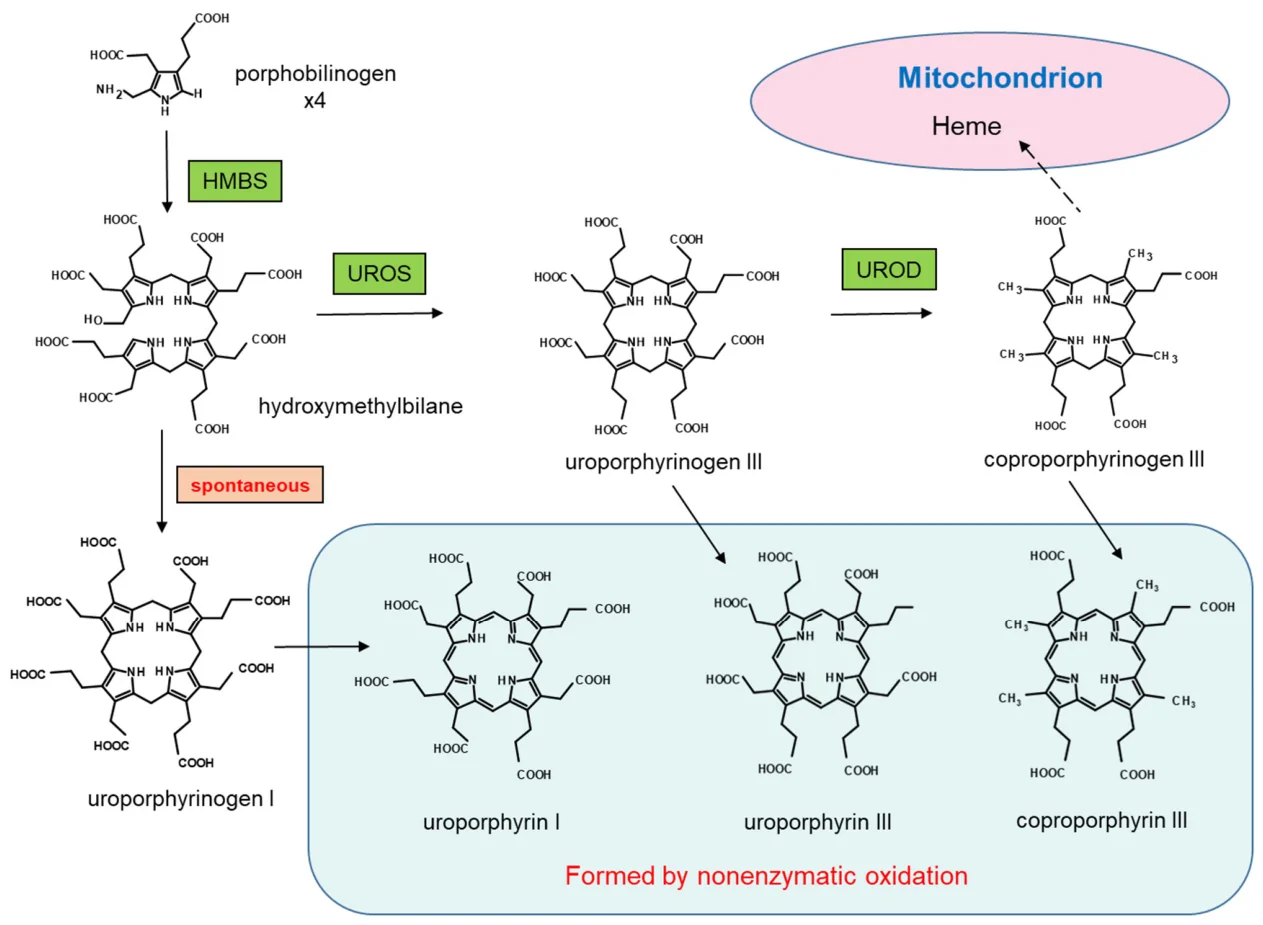
Oxidation of hydroxymethylbilane and uroporphyrinogens to uroporphyrins. The porphyrins are excreted in the urine but can be deposited in the liver and other tissues causing them to fluoresce under Wood’s lamp UV light and in skin causing phototoxic damage after exposure to sunlight.

Mechanisms of oxidation of the cytosolic porphyrinogen products, particularly uroporphyrinogens, and cellular protection, are less understood than over production of protoporphyrin IX. Some of these aspects occur in human and experimental disorders of heme synthesis in which a massive accumulation of uroporphyrins occur as a consequence of uroporphyrinogen oxidation. In the very rare cutaneous genetic disorder congenital erthropoietic porphyria (CEP), mutations of the *UROS* gene cause uroporphyrin I accumulation, predominantly in the bone marrow, and urinary excretion. In the most common porphyria, sporadic porphyria cutanea tarda (sPCT), uroporphyrin levels in the liver, skin and urine are greatly elevated alongside depressed hepatic activity of UROD (Figure 1 and Figure 2). sPCT has both hepatic and subsequent cutaneous injury triggered by a variety of other factors, including iron metabolism and individual susceptibility. Certain chemicals, such as hexachlorobenzene (HCB) and 2,3,7,8-tetrachlorodibenz-*p*-dioxin (TCDD), cause a similar effect to sPCT in both people and animals and is a well reported toxicological hazard considered in risk assessment (Table 1) [14].

In the following Sections, the development and present knowledge of the field of uroporphyrinogen oxidation, susceptibility and outcomes are reviewed. In particular, a number of areas are highlighted that are puzzling and require further investigation to explain fully the mechanisms of some human porphyrias and overlap with uroporphyria caused by chemicals of toxic concern. Other details of the clinical biochemistry and pathobiology of sPCT and the pertinent toxicology of HCB and TCDD can be found elsewhere [8,14,15,16,17,18].

## 2. Hydroxymethylbilane and Cytosolic Porphyrinogen Oxidation

### 2.1. Oxidation to Porphyrins

It took many years to understand the polymerisation of four porphobilinogen units to form hydroxymethylbilane (HMB) catalysed by hydroxymethylbilane synthase (HMBS) and subsequent cyclisation to the asymmetric intermediate uroporphyrinogen III (Figure 2) [1]. Two condensed porphobilinogen molecules covalently attached to the enzyme near the active site, act as a cofactor for the linear addition of four additional pyrroles releasing HMB on hydrolysis and regenerating the dipyrrole cofactor [19]. Importantly, the tetrapyrrole is not particularly stable. It will form the symmetrical macrocylic uroporphyrinogen isomer I spontaneously by elimination of water in conditions compatible with the cellular environment (Figure 2) [7]. However, the ultimate structure of biological heme, requires the intermediacy of the asymmetric isomer III. Uroporphyrinogen I does not isomerise to uroporphyrinogen III [20]. Instead, in vivo HMB is converted to uroporphyrinogen III by uroporphyrinogen synthase (UROS) via an intra spiro rearrangement and at a faster rate than the spontaneous cyclisation to uroporphyrinogen I [1,21]. UROS is less stable than HMBS and prior heating of erythrocyte lysates and incubation with porphobilinogen leads to the accumulation of uroporphyrin I (after oxidation), not uroporphyrin III [22,23].

The carboxyl groups of the four acetate side chains of uroporphyrinogen III are removed by uroporphyrinogen decarboxylase (UROD), to form coproporphyrinogen III (Figure 2) [24]. Only coproporphyrinogen III is processed further to heme after transportation into the mitochondrion. In some human disorders and in experimental porphyrias all heptacarboxylic, hexacarboxylic and pentacarboxylic intermediates during UROD action can be released and oxidised to their porphyrin analogues [25,26,27]. The first product, heptacarboxylic porphyrinogen III*d* seems to be particularly prone to accumulate under conditions of higher substrate concentration or depleted UROD activity [28]. The reason for this is not clear but as it does not happen with uroporphyrinogen I it is probably a consequence of the inverted position of the D ring CH_2_COOH group in the III isomer. Although decarboxylation by UROD of uroporphyrinogen I to the respective porphyrinogen I isomers does occur, coproporphyrinogen I is not utilised. Chemically the cytosolic porphyrinogens are more stable in acidic conditions than alkaline but all may be oxidised to the porphyrin analogues in vitro and in vivo.

After oxidation, the high levels of uroporphyrinogens produced by deficits of UROS and UROD activities in CEP and sPCT patients, respectively, lead to large quantities of the uroporphyrins excreted in urine (Figure 2). Massively elevated levels of the porphyrins are also found in excreta from humans and rodents exposed to HCB [14,25,28,29]. Even so, at normal levels, in metabolic disorders and experimentally, both porphyrinogens and porphyrins can be present in tissues and urine [27,30]. The main urinary porphyrins derived from uroporphyrinogens in these disorders or poisonings are uroporphyrin I in CEP and uroporphyrin I, uroporphyrin III and heptacarboxylic porphyrin III*d* in sPCT but also small amounts of the coproporphyrin isomers I and III depending on the scenario involved (Figure 2) [31]. Although in clinical biochemistry the uroporphyrins are considered as side oxidation products of the porphyrinogens, their presence as colours in birds and marine shells demonstrates they can have other roles in nature [32,33].

**Figure 3:**
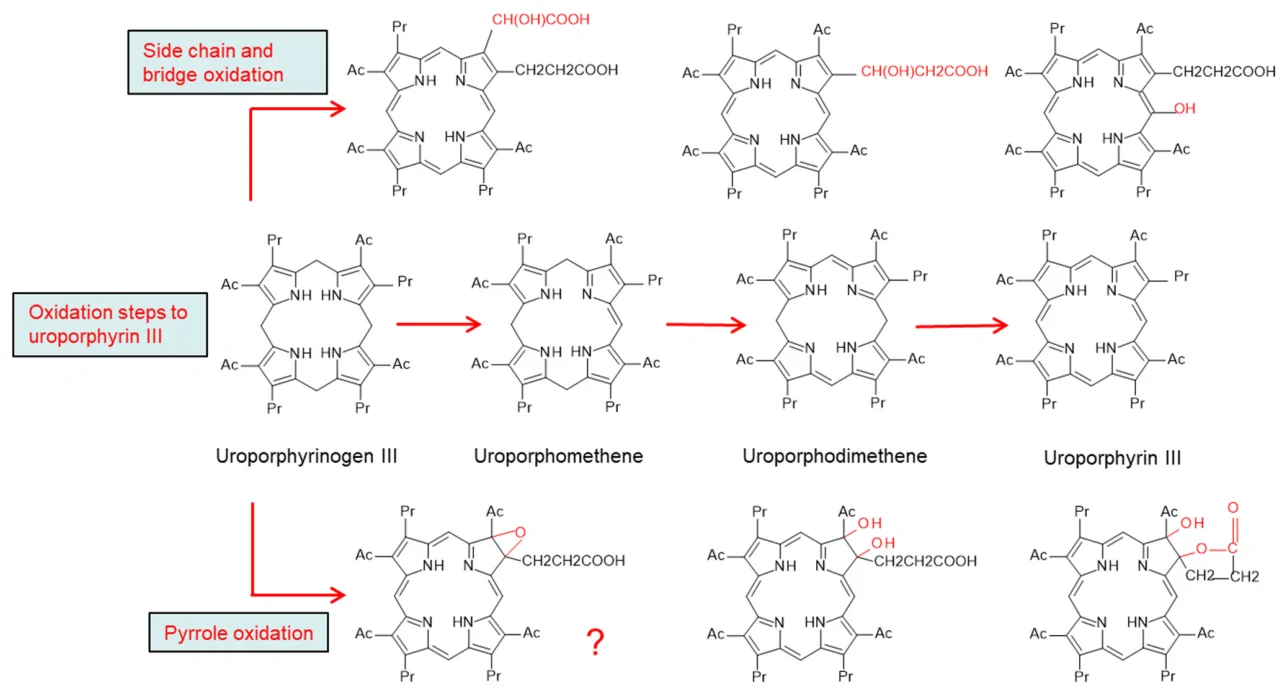
Intermediates in the oxidation of uroporphyrinogen III to uroporphyrin III and some identified additional oxidation products. A similar range of products is possible in the oxidation of uroporphyrinogen I and heptacarboxylic porphyrinogen III*d* as well as other cytosolic porphyrinogens. Oxidation side products have been detected in urine and in experimental liver except for the epoxide, which is a postulated intermediate [22,39,40,41,42,43,44]. Red color indicates groupings with oxygen addition and ? not proven product.

The oxidation of uroporphyrinogens to uroporphyrins involves the withdrawal of six electrons via the partially oxidised intermediates uroporphomethene and uroporphodimethene (Figure 3) [20,24,34]. It would be interesting to know the degree and time of their occurrence intracellularly and any difference between hepatic and nonhepatic tissues.

### 2.2. Oxidation to Hydroxylated Porphyrins and Nonporphyrin Products

In early studies of uroporphyrinogen and uroporphyrin, the formations of nonporphyrin molecules derived from uroporphyrinogen were noted. Absorption spectroscopy of a green pigment was compatible with chlorins formed by oxidation but techniques were not advanced enough for characterisation [24,32,35]. Oxidation of uroporphyrinogens (and HMB) in the dark by an accepted mild enzymic O_2_^−·^ generating system causes a slow conversion to uroporphyrins but in the presence of Fe-EDTA the rate of oxidation is enhanced markedly consistent with forming the more potent HO^.^ radical oxidising species [36]. A significant amount of products formed (approximately 50%) are not porphyrins, with loss of Soret absorbance, and cannot be converted back to uroporphyrins. A different system in vitro with H_2_O_2_ or liver microsomes and Fe-EDTA gives similar findings [37]. Lin and Timkovich characterised by NMR, mass spectrometry and spectroscopy a wide variety of hydroxylated porphyrins, chlorins, and other oxygenated tetrapyrroles produced from uroporphyrinogens and coproporphyrinogens during oxidation in the air and dim light. Possibly singlet oxygen and trace amounts of products acted as photocatalysts [38]. There is limited information on the presence of these in vivo under normal and porphyric conditions, probably because of the difficulty in detection. Representatives of hydroxylation of acetate and propionate side chains, hydroxylation of the macrocyclic ring at a *meso* bridge position, dihydroxy pyrrole groups and chlorins are illustrated in Figure 3. Potentially in vivo, there are many more compounds depending on the presence of uroporphyrinogen I, uroporphyrinogen III and heptacarboxylic porphyrinogen III, as well as coproporphyrinogens. Formation of the diol and chlorin from uroporphyrinogen was postulated to occur via initial epoxides on a pyrrole. Some of the hydroxylated products derived from uroporphyrinogens are also detectable by HPLC-mass spectrometry in the urine or liver of patients with CEP and PCT and rats with porphyria caused by HCB [22,39,40,41]. Chlorins were identified in later investigations using more sophisticated mass spectrometry in the liver of porphyric mice treated with TCDD and after photochemical oxidation of uroporphyrinogen I, but not epoxides or peroxylated derivatives that were reported in earlier studies [42]. The potential to create artefacts in biological-derived material and changes in proportions of oxidative products depending on the conditions means that analysis of uroporphyrinogen oxidation products from in vitro and in vivo sources has to be performed with great care. References cited above describe more details of the mechanisms and methods of identifications. Whether any of the oxidation products of uroporphyrinogens have biological properties is an interesting thought perhaps worthy of investigation.

### 2.3. Oxidation of Uroporphyrinogens to Uroporphyrins in Human Disorders

The very rare cases of patients with mutations of the *UROS* gene causing the severe cutaneous condition CEP are characterised by extreme photosensitivity of the skin due to the deposition of high levels of mostly uroporphyrin I causing marked sensitivity to sunlight with fragility and blisters of the skin of affected individuals [8,23]. The primary source of the uroporphyrin appears to be uroporphyrinogen I formed spontaneously from accumulating HMB due to compromised UROS in the erythropoietic system of the bone marrow. As referred to above, minor further oxidation products of uroporphyrin I in urine have also been detected, presumably formed at the uroporphyrinogen stage [39,40,41,42,43,44].

Clinically, the most common incidence of uroporphyrinogen oxidation is the hepatic disorder sporadic porphyria cutanea tarda (sPCT) mostly occurring in middle-aged to older people [8]. This disorder is characterised by marked inhibition of hepatic UROD with very high levels of uroporphyrins I and III in the liver and in urine, and with mild to moderate liver injury. Liver biopsies fluoresce bright red under Wood’s UV-A light due to the presence of uroporphyrins with characteristic birefringent needle-shaped crystals [45]. As in CEP these porphyrins are deposited in the skin making it photosensitive with fragility, blistering, pigmentation, scarring and hypertrichosis, and in other tissues, such as teeth. sPCT is considered an acquired disease. A pathogenic mutation of *UROD* is not a prerequisite for the development of sPCT, which is usually associated with alcohol consumption, tobacco use, estrogenic drugs or certain viral infections, such as HIV and hepatitis C [46]. A variant of *UROD* can be a risk factor in a familial form of the disorder (fPCT) [8]. The link between some aspects of iron metabolism and the other triggering factors of sPCT, such as hepatic viral infection, is well established and partially have been mechanisms proposed [46]. Iron stores per se may be mild or moderate but are not predictive of the disorder. Lowering hepatic iron levels by phlebotomy or administration of desferrioxamine causes remission [8,16,46,47,48,49,50,51,52,53]. In populations of European descent, inheritance of the pathogenic variants of the haemochromatosis gene (*HFE*) can be one major risk factor but not necessarily elsewhere for other patients [15,48,54]. Much evidence suggests that the oxidation of uroporphyrinogen to uroporphyrin is central to the mechanism of UROD inhibition [14,55,56].

A severe disorder resembling sPCT was reported in the 1950/60s after an episode of poisoning in Türkiye following prolonged contamination of food by the fungicide HCB [29,54,57,58,59]. This is still considered an important episode in human toxicology. There is no evidence that in the Turkish PCT-like disorder the patients had elevated levels of iron and it is presumed that hepatic UROD was inhibited. Co-exposure to other chemicals may have compounded the problem. Porphyria after exposure to TCDD in the work place in USA and Europe has been reported but the evidence is not robust that uroporphyria is a common response in humans to this chemical [14,18,60,61,62,63]. Follow-up studies of HCB affected individuals showed that the metabolic effect could persist for many years [64,65]. As with sPCT, the evidence was compatible with over production of uroporphyrinogens and their oxidation to uroporphyrins. Exposure at occupational/environmental levels of these chemicals probably have little significant porphyrogenic effect [66,67].

Reports of mildly raised levels of uroporphyrins and particularly coproporphyrins in the urine after low exposure to various metals, such as mercury, and to other chemicals do not seem to be due to overt disease states but may reflect changes in metabolism and oxidative environment. Some of these may be variation in kidney function. Many studies have also linked elevations to incidence of autism although this requires a lot of systematic critical examination [31,68,69].

### 2.4. Experimental Studies of Mammalian Uroporphyria

**Figure 4:**
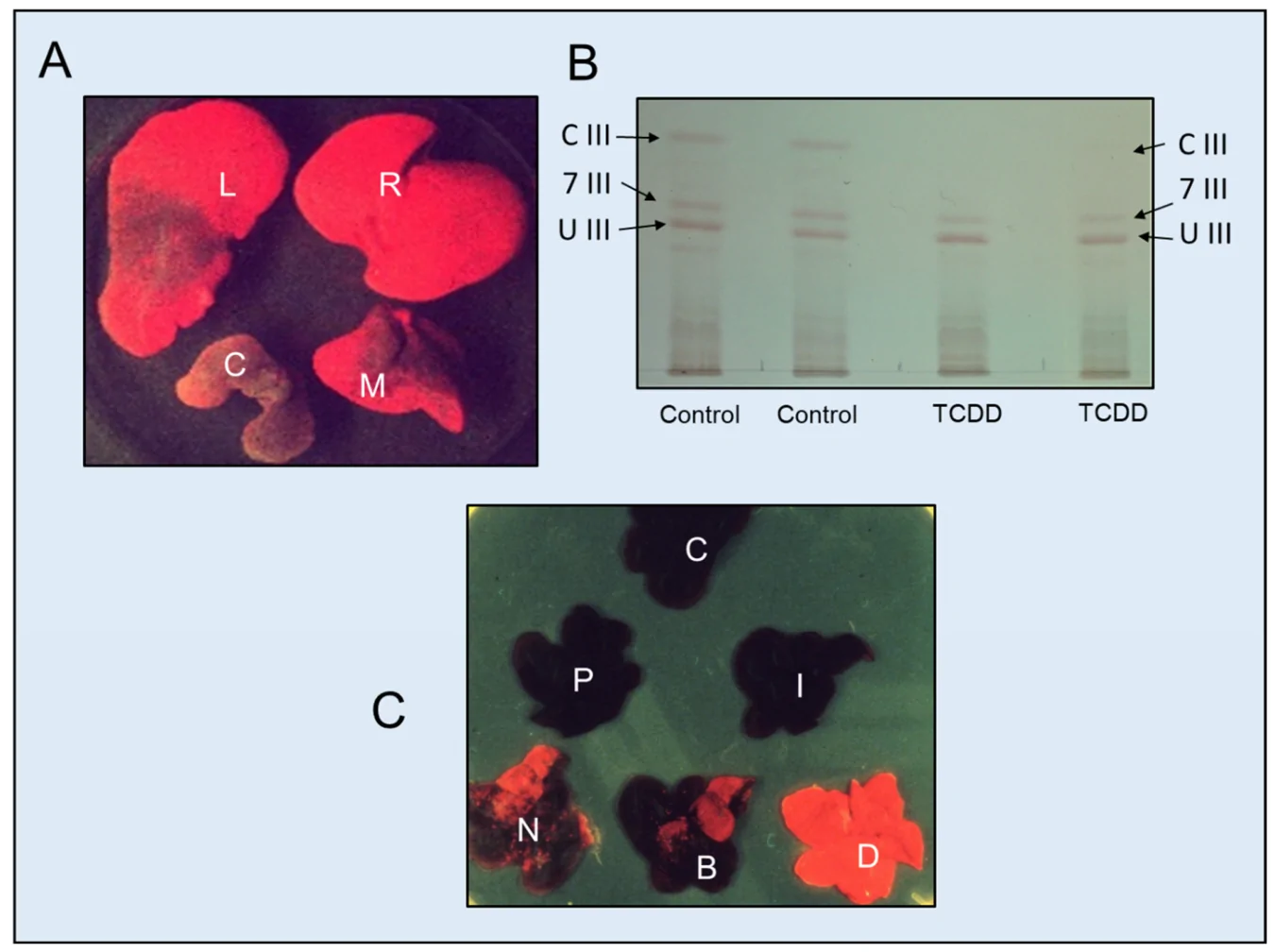
Some uroporphyric responses after in vivo experiments. (**A**). Variable fluorescence under Wood’s UV light of lobes of liver from female rats after consuming hexachlorobenzene (HCB). L, left; R, right; M, median; C, caudate [87]. It also demonstrates discrete foci of porphyria. HCB levels were not significantly different. (**B**). Example of inhibition of UROD in liver from C57BL/10ScSn mice treated with the potent AHR ligand 2,3,7,8-tetrachlorodibenzo-*p*-dioxin (TCDD). Decarboxylation of uroporphyrinogen III (U III) by liver supernatant from control or TCDD treated mice was estimated by micro thin layer chromatography of porphyrin methyl esters following mild iodine oxidation of porphyrinogens and esterification of resulting porphyrins. Decarboxylation to coproporphyrinogen (C III) was decreased markedly by TCDD treatment but the heptacarboxylic porphyrinogen (7 III) showed some accumulation as in control [90]. (**C**). Fluorescence of iron loaded C57BL/10ScSn mouse livers after prolonged dietary exposure to nonchlorinated polycyclic hydrocarbon ligands of AHR. C, control; P, phenobarbital; I, isosafrole; N, β-naphthoflavone; B, benz[*a*]anthracene; D, dibenz[*ah*]anthracene [91]. A version was published previously in black and white [92].

Following the reports of HCB causing a sPCT-like disease in people, experimental studies demonstrated that the hepatic porphyric responses in particular could be reproduced in rats sometimes taking many weeks [70,71,72]. A variety of studies demonstrated the accumulation of uroporphyrins I and III in the liver and urine as well as the partially decarboxylated intermediates especially hepatacarboxylic porphyrin III*d*. Iron overload potentiates the porphyrogenic effect of the chemical and desferrioxamine may modify the response as in sPCT [70,71,72,73,74,75,76,77,78,79,80,81,82]. UROD is markedly inhibited. The greater sensitivity of female than male rats to HCB and effect of estrogenic drugs could be due to higher hepatic iron levels and turnover in the former [83,84,85]. As had been observed from human biopsies, the development of porphyria in the liver is not at first uniform with initial centrilobular (perivenous, zone III) fluorescent foci [78,86]. The lobes of the liver respond at different rates that may be due to variable induction of cytochrome P450 or levels of ROS and protection enzymes (Figure 4A) [78,87]. Initial macroscopic examination of the livers show particular regions that are highly fluorescent under UV light but more of the liver becomes fluorescent within a short time after exposure to visible light suggesting that appreciable amounts of porphyrinogens were originally present (20–40%) [27]. TCDD also causes porphyria in female rats after multiple doses up to at least 16 weeks with a slow recovery rate [88,89].

C57BL/6J mice are responsive to µg/kg doses of TCDD causing porphyria, (even a single dose), with marked inhibition of the decarboxylation of uroporphyrinogen III to coproporphyrinogen (Figure 4B) [90,93,94]. The porphyric response in C57BL/6 mice after TCDD leading to accumulation and oxidation of uroporphyrinogen appeared to be inherited with inducibility of the microsomal cytochrome P450 1A1/2 enzymes regulated by the aryl hydrocarbon receptor (AHR) for which the chemical is a high affinity ligand [94,95]. Importantly, lowering hepatic iron levels in mice by phlebotomy or low iron diets gives some protection from both uroporphyria and liver damage whereas a predose of iron to C57BL mice strongly enhances the response [96,97,98].

Unlike TCDD, early studies seemed to show that mice were insensitive to HCB causing porphyria despite fatty livers and neurotoxicity. However, prior iron overload of C57BL/10ScSn mice not only enhances the TCDD-induced uroporphyria, but causes a surprisingly strong uroporphyric response to HCB and polychlorinated biphenyls (PCBs) [99,100,101]. Since induction of CYP1A1/2 by the chlorinated chemicals via the AHR seemed implicated in the uroporphyric response, it seemed logical to determine whether classical nonchlorinated inducers acting via the AHR, such as β-naphthoflavone and 3-methylcholanthrene, could also cause porphyria in mice. This hypothesis was proved correct (Figure 4C) [91]. Some non-AHR inducers of cytochrome P450, phenobarbital and acetone, were also effective, but only weakly. Co-administration of 5-ALA in the drinking water greatly speeds up the response [102,103]. The combination of ethanol in drinking water and iron loading due to knockout of *Hfe* in one strain of mouse produced uroporphyrin and UROD inhibition after 4–6 months [104].

In all the earlier experimental studies, the influence of iron was presumed to be dependent on co-exposure to the inducers of the microsomal P450 system [91,102]. Even 3 months after iron overload (which persists throughout life) little effect on uroporphyrin excretion occurs in mice that have only received iron [91]. Despite this assumption, following a longer delay period (approximately 5–6 months), C57BL/10ScSn and SWR mice eventually do develop uroporphyria after iron overload alone. The response is accelerated by imbibing 5-ALA [99,105,106,107]. Curiously, in contrast to other inducers of cytochrome P450, administration of nafenopin a CYP4A inducer and peroxisome proliferator is protective [108,109]. Surprisingly without any additional administration of iron, a mild hepatic uroporphyria develops in SWR mice after consuming 5-ALA alone for 8 weeks [110].

These stepwise findings of the factors inducing uroporphyria and associated inhibition of UROD in experimental porphyria caused by chemicals, demonstrate that the process is multifactorial. It is also evidence for a common fundamental mechanism. A number of different stimuli acting together can cause a very severe but rare response. This is an important, more general concept in the idiopathic toxicity of drugs and chemicals.

## 3. How Is Hepatic Uroporphyrinogen Oxidised to Uroporphyrin?

### 3.1. Oxidation of Uroporphyrinogen by Free Radical Generating Systems

Oxidation of uroporphyrinogens to their respective uroporphyrins has been explored in a number of simple systems involving ROS as models of what might occur at a molecular level [36,37,111,112,113,114]. In some of the more chemical studies, iron potentiates the rate of oxidation to uroporphyrin whereas glutathione protects. With one system the yield of uroporphyrin from the porphyrinogen based on recovered uroporphyrin content by absorption spectroscopy or HPLC was markedly reduced, indicating that products other than porphyrins are formed [36]. The possibility of the presence of uroporphomethene during oxidation has been detected in some experiments.

### 3.2. Oxidation by Cytochrome P4501A2

Many investigations in understanding the uroporphyria of sPCT and that caused by chemicals have focussed on the oxidation of uroporphyrinogen to uroporphyrin using cellular, microsomal or rapid in vitro avian models. The microsomal component CYP1A2 has been explored because of the association of porphyria development in rodents with its induction by ligands of the AHR, such as 3-methylcholanthrene and TCDD-like polychlorinated biphenyls [14]. A plethora of different in vitro systems have been employed, including sensitive cultured fish and chick embryo liver cells and cultured mouse hepatocytes [111,115,116,117,118,119,120,121,122,123,124,125,126,127,128,129,130]. The focus here is on mammalian systems; other studies are listed in the references of cited papers. Findings can seem confusing, including whether oxidation is accompanied by inhibition of UROD. In some studies the presence of some forms of iron enhanced the oxidation of uroporphyrinogen to uroporphyrin by mouse microsomes and reconstituted CYP1A2. Other experiments concluded that the mechanism is not free radical and that the porphyrinogen binds to the active site of the cytochrome P450 [131]. There is an apparent difference on a requirement for co-binding of chlorinated chemicals, such as TCDD-like PCBs between microsomes and cytochrome P450 from avian species and those from rodent species. Studies of liver microsomes from people and the expression of CYP1A2 in cell systems, suggest that the human enzyme can catalyse oxidation of uroporphyrinogen. It is much less active than the mouse cytochrome. Importantly for sPCT, initially most of the microsomal oxidation of porphyrinogens in humans is not accounted for by CYP1A2 content. Over a long period of the development of the disorder it is possible that the involvement of CYP1A2 becomes more important [132,133,134].

**Figure 5:**
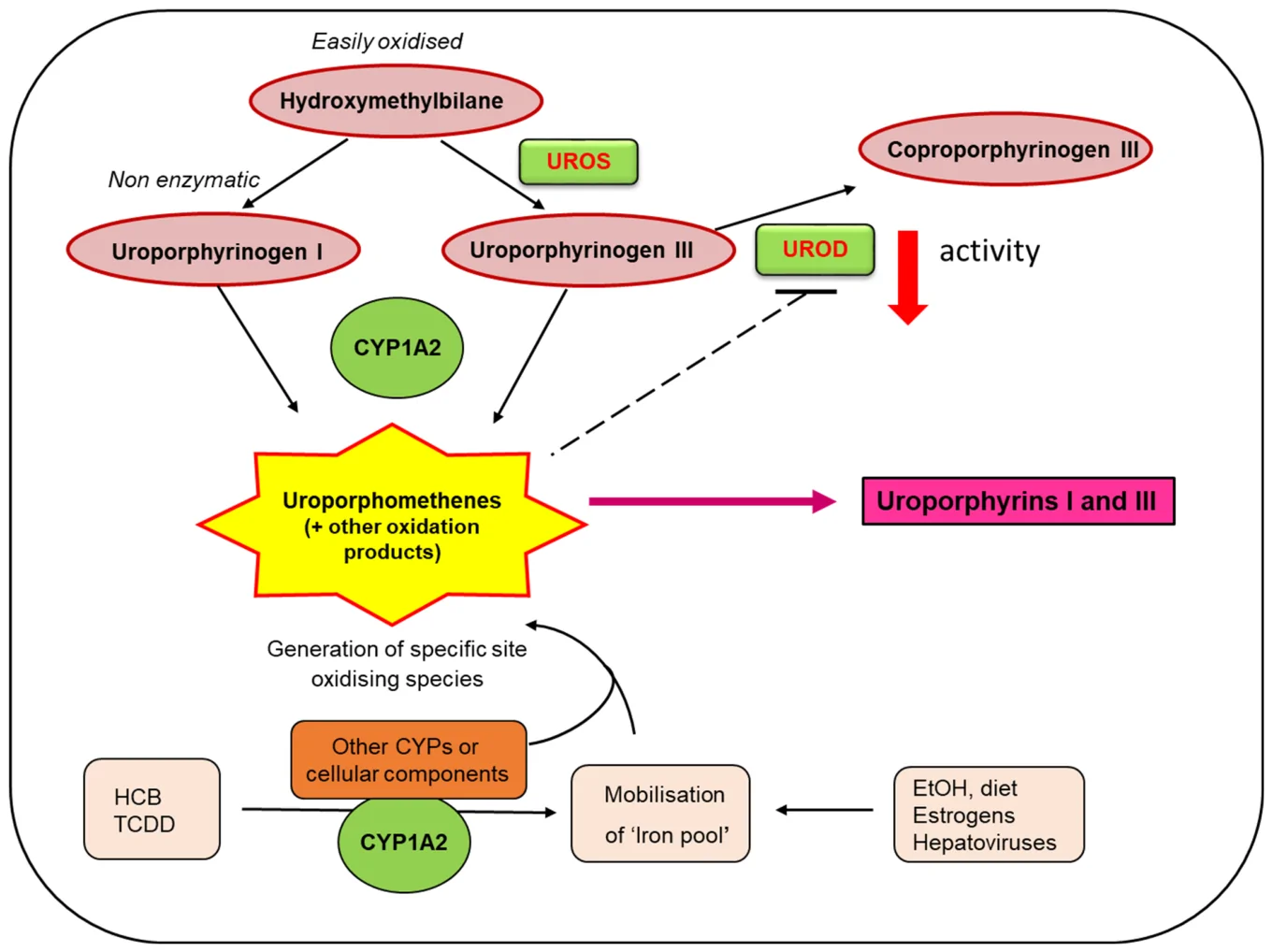
Schematic hypotheses leading to the oxidation of uroporphyrinogen in vivo. Although there is considerable evidence for a role of CYP1A2 in experimental models, and the influence of iron homeostasis in humans and animal experiments is well established, the details are still not resolved, despite assumptions and assertions to the contrary. See also [46].

One of the implications from in vivo mouse models is that induction of cytochrome P450 is not essential and it may be that only small constitutive levels of CYP1A2 above a threshold are required to produce a uroporphyrogenic response [103,107,110,135,136,137,138]. The introduction of gene knockout technology in mice allowed a different in vivo approach to investigate the involvement of CYP1A2. C57BL/6 mice null for the *Cyp1a2* gene do not develop uroporphyria whether initiated by iron/5-ALA, HCB or TCDD, at least under the conditions reported [137,139,140,141]. The results from these experiments seem clear, no CYP1A2 no porphyria even with strong expression of the related enzyme CYP1A1 induced by TCDD, and does not depend on the laboratory source of the knockout mice. However, curiously in *Cyp1a1* (−/−) null mice significant protection is observed [142]. By combining a variety of genetic, iron and inducing factors, uroporphyria is ultimately possible in *Cyp1a2* (−/−) mice perhaps illustrating that there may be different mechanisms of uroporphyrinogen oxidation (Figure 5) [46,56].

The proposal that threshold levels of CYP1A2 are crucial in experimental uroporphyria raises a number of uncertainties. An appreciable proportion of the oxidation of uroporphyrinogen to uroporphyrin measured in microsomal systems seems to be due to other CYP enzymes or processes, even in knockout mice. When inducers of CYP1A2 are given in vivo to elevate uroporphyrinogen oxidation that is subsequently measured in vitro, increases have been modest especially when compared with increases in CYP1A2 protein and particularly in activity with model substrates, such as methoxyresorufin. Why would an endogenous relatively hydrophilic octacarboxylic porphyrinogen be a substrate for a CYP enzyme embedded in the lipophilic environment of the microsomal system?

The ‘natural’ substrates and role of hepatic CYP1A2 are not understood fully but are clearly complicated and may be unexpected [143]. It has been proposed that CYP1A2 acts as an electron ‘sink’ from other cytochrome P450s and that TCDD and related chemicals generate reactive oxygen species from liver microsomes and mitochondria by uncoupling, which may be pertinent to oxidation of uroporphyrinogens [120,144,145]. What may be relevant in this respect is the demonstration that mouse and human CYP1A2 is the main protein binding site and reservoir in the liver for TCDD and related chemicals [142,146,147,148,149,150]. Why this occurs, rather than other cytochrome P450 species (e.g., CYP1A1) is unclear. The influence on the action of CYP1A2 in uroporphyria development could be related to the binding of these chemicals causing uncoupling. However, Jacobs et al. reported that oxidation of uroporphyrinogen by mouse microsomes only occurs in the absence of any inducer remaining with CYP1A2 [122]. Interestingly, with avian and mouse preparations, ascorbic acid seems to afford some protection against the oxidation of uroporphyrinogen to uroporphyrin, including the intermediacy of uroporphomethene (Figure 3) [151]. It might prove surprising and interesting to investigate porphyria development with other CYP null mice. Perhaps the involvement of the microsomal system is more complex than we envisage.

## 4. Inhibition of Uroporphyrinogen Decarboxylase In Vivo

As well as high accumulation and excretion of uroporphyrins I and III by oxidation of the uroporphyrinogens, sPCT and experimental porphyrias in rodents are characterised by marked depression of hepatic UROD activity. The exact relationship between these two phenotypes is unclear. Inhibition of UROD likely causes a pool of uroporphyrinogen III susceptible to oxidation when UROS is fully active. Human UROD is a dimeric enzyme and autosomal dominant heterozygosity of pathogenic variants, with 50 percent loss of activity in all tissues, contributes to fPCT and homozygosity or compound heterozygosity, to the very rare disorder hepatoerythropoietic porphyria (Table 1) [152,153,154,155,156]. A partial model of fPCT using mice heterozygous null for *Urod* shows approximately 50% activity in all tissues prior to experiments to produce porphyria [157,158,159,160]. In sPCT the activity of UROD is markedly depressed (below 50%) only in the liver, without loss of immunogenicity, during phases with active skin lesions but returns to normal in remission [161,162,163,164,165,166]. In rats and mice after exposure to HCB and TCDD, uroporphyria develops following a delay period and hepatic UROD activity is depressed significantly as a gradual depletion from 100% without loss of immunogenicity [28,73,75,77,82,90,94,100,161,162,163,164,165,166,167,168,169,170]. Early proposals suggested that a metabolite of HCB or of other slowly metabolised chlorinated chemicals caused inhibition of UROD. Later, this explanation became untenable [82,84,129,171,172,173,174,175]. This always seemed unlikely because of the very slow metabolism of these chemicals, the very small doses of TCDD required, the demonstrations that uroporphyria can also be caused by nonchlorinated aromatic chemicals and that iron overload eventually causes the effect [91,93,99,102,106].

An alternative hypothesis proposed that sPCT and chemical-induced porphyria share a common mechanism associated with the oxidation of uroporphyrinogens, perhaps diverging in the initial stages [55]. Heat stable fractions from porphyric livers inhibit UROD [113,174,176,177,178,179]. Based on the original investigations of UROD, the tetra- and dihydroporphyrin intermediates in the oxidation of uroporphyrinogens (Figure 3) were suggested as possible inhibitors and could be detected in vitro prevented by ascorbic acid [24,118,151,180]. A uroporphomethene was identified in such fractions as a competitive inhibitor of mouse and recombinant human UROD [181]. The analytical chemistry basis for this identification has been questioned and perhaps the original extracts require further examination and confirmation [182,183]. Inhibition of UROD by such tetrapyrroles is defensible particularly as the porphyrin analogues have inhibitory properties [27]. However, there are inconsistencies in the studies of inhibitory extracts from HCB and TCDD treated liver and the study reporting the identity as a uroporphomethene [113,174,176,177,178,179]. In the earlier studies, the inhibitor was not competitive, stable to heating at up to 100 °C for 30 min, could be purified through SEP-PAK C_18_, stored in the freezer for long periods and though dialysable could not be reversed after incubation with the enzyme source. With pentacarboxylic porphyrinogen I as substrate, UROD was inhibited by >90% with evidence that significant inhibition occurred before a major rise in hepatic porphyrin levels [175,179]. Are these findings completely compatible with uroporphomethene as the inhibitor? Is the inhibitory fraction from porphyric livers the prime reason for UROD insufficiency and maintained inhibition? Detailed chemical studies of the oxidation and reduction in uroporphyrinogens with respect to oxygen, photosensitivity and temperature, have been described but it is not clear if these are pertinent to the stability and reactivity of uroporphomethenes in physiological environments [20,24,34,175,179].

A preliminary report suggested partial oxidation during nonenzymatic cyclisation of HMB to uroporphyrinogen I is the first step in the porphyrogenic sequence leading to uroporphomethene I as a UROD inhibitor [184]. Further oxidation to uroporphyrin I could explain why the isomer is often found in uroporphyric urine and liver in sPCT and experimental studies in a greater proportion than the III isomer. This might be true but in mice, the relative hepatic levels of the isomers change with development of porphyria and the III isomer predominates at a very early stage [175]. Of course oxidation of the two isomers may be synchronous and not mutually exclusive. It might be expected that HMB is similarly oxidised to uroporphyrin I intracellularly [36]. UROD inhibition is not a reported characteristic of CEP but this might reflect that the disorder is erythropoietic mainly originating in bone marrow, and not in the physiologically different environment of the liver [185]. Interestingly from prior siRNA and modelling experiments, a synthetic porphodimethene inhibiting UROD has been explored as an anticancer agent [186,187]. The oxidation of uroporphyrinogens also leads to nonporphyrin tetrapyrroles, such as chlorins, that have been detected in vivo and in urine or plasma from sPCT and CEP patients (Section 2 and Figure 2) [22,36,38,39,40,41,42,43,44]. Whether these further oxidation products of uroporphyrinogen in vivo are of any importance in inhibiting UROD, or as markers of another inhibitory process, is unknown although causing modest inhibition in vitro [36].

The handling, mechanism and sequence of decarboxylation of uroporphyrinogens by UROD have often been explored [1,25,188,189,190]. Unlike most decarboxylases UROD does not have a cofactor’ [191]. A consistent observation is that with inhibited hepatic enzyme or high substrate concentrations in vitro, heptacarboxylic porphyrinogen III accumulates (e.g., Figure 4b). The *d* isomer (i.e., removal first of the acetate carboxyl on the asymmetric D ring) predominates over the other three isomers but may be random in erythropoietic cellular environments [190,192]. This accumulation before decarboxylation to the hexacarboxylic porphyrinogens may be pertinent to the mechanism of UROD insufficiency in uroporphyria, particularly since hepatacarboxylic porphyrin III*d* is the most inhibitory of the porphyrins [27]. An interesting possibility would be suicidal inactivation of the enzyme at the active site in the attempted decarboxylation of a uroporphomethene.

As might be expected, UROD activity is markedly diminished by exposure to light in the presence of uroporphyrins, presumably by deactivation at the active binding site, demonstrating the possibility of site directed inhibition in vivo [179,193,194]. Mechanistically, all of the above experimental findings need to be explained from the perspective of the oxidation of uroporphyrinogens to uroporphyrins by rodent CYP1A2 and the resistance of the *Cyp1a2* null mouse (Section 3.1). Could HMB be a substrate for CYP1A2 [195]? In addition, there is also a variety of associated factors, including iron metabolism and genetics, that have significant influence on the acquired nature of sPCT and the experimental uroporphyria in animals, that probably give insights in to the mechanism. Ideally, if the inhibition of UROD from in vivo models is due to a competitive inhibitor, restoration of full enzyme activity should be possible and the identification of uroporphomethene confirmed. If not, the isolation and characterisation of inhibited enzyme could be undertaken.

## 5. The Involvement of Iron

A multitude of studies of human diseases have implicated or explored the potential involvement of iron catalysing ROS formation leading to oxidative pathological lesions [196]. Although not often quoted, the association of primary haemochromatosis with sPCT is a very good example of this [16]. Experimentally, many studies have shown hepatic intracellular oxidative endpoints that are catalysed by iron in HCB and TCDD treated rodents. Whether these nonspecific effects relate to the oxidation of uroporphyrinogens and the inhibition of UROD is unclear [134,144,197,198,199,200,201,202,203,204,205,206,207,208]. Vitamin E and ascorbic acid have some protective effects [136,151,209,210,211]. The demonstration that iron stores markedly affect the hepatic toxicity of TCDD, particularly porphyria, in C57BL/6J mice was a significant finding for experimental design. It culminated in the simple demonstration that a minimum level of elevated iron stores alone is eventually porphyrogenic. The enhancement by consumption of the heme precursor 5-ALA, probably creates a larger pool of heme intermediates (Section 2.4) [97,98,99,105,106,107,136,212,213]. Thus the oxidation of uroporphyrinogens leading to uroporphyria can be regarded as one of many aspects of the toxicity of iron enhanced by a variety of other factors. Many of those triggering factors associated with sPCT, such alcohol and hepatic viral infections, are associated with modulation of iron metabolism [46].

Investigating the exact role of iron experimentally has proven challenging, especially because of the delayed response and difficulty of reproducing and studying this with in vitro mammalian models [116,127]. The possibility that Fe^2+^ directly inactivates UROD, perhaps by oxidising cysteines, is not considered likely, partly due to the concentration of free iron required [134,214,215,216,217]. Suggestions that the mechanism of iron is by upregulation of *ALAS1* is not supported by experiments, including co-administration of 5-ALA [105]. One obvious and popular possibility is that it catalyses a particular form of ROS, perhaps hydroxyl radicals, speeding up the oxidation of uroporphyrinogens, but there is no consensus on how. As described earlier, in rodents at least, there is evidence that CYP1A2 may play a role oxidising uroporphyrinogens completely to uroporphyrins. This oxidation has been described as taking place wholly within the cytochrome environment and not the equivalent of experiments with iron-catalysed enzyme or microsomal ROS generating systems oxidising uroporphyrinogens [131,218]. However, if uroporphomethenes are formed that inhibit UROD it would require their ‘leakage’ from CYP1A2 before complete oxidation to uroporphyrin. What is the evidence for this scenario and what then would be the action of Fe^2+^? Initial porphyria in zone III of the liver lobule could support the involvement of CYP enzymes and iron and perhaps GSH protection, although this is at the lowest level of oxygen tension.

Alternative systems for uroporphyrinogen oxidation that involve iron have been proposed but have to take into account the protective effect in mice of knocking out the *Cyp1a2* gene [56,92,140]. In some systems rapid in vitro oxidation of uroporphyrinogen (or HMB) to uroporphyrin appears to require stronger oxidants than O_2_^−·^ or H_2_O_2_. In other studies the opposite has been reported [36,92,112,118]. Oxidation should be slow or mild enough for uroporphomethene to accumulate and act as an inhibitor of UROD. The potential disproportionation of the dihydroporphyrin to uroporphyrin and the tetrahydroporphyrin could be important in this respect [34]. Since in model studies of uroporphyrinogen oxidation catalysed by Fe-EDTA, tetrapyrrole-type oxidation products are formed that cannot reform uroporphyrins, as described in Section 2.2, further investigation of uroporphomethene in the presence of iron-catalysed conditions might prove informative [36,113,118].

Where and what are the forms of iron taking part in the uroporphyric response in mammals? Release of ferrous iron from ferritin and other sources by liver microsomes and by routes pertinent to porphyria and hepatic toxicity have been explored but whether these are intracellularly relevant remains an open question [219,220]. Although ferrous iron (Fe^2+^; Fe(II)) is frequently quoted as taking part in Haber–Weiss and Fenton reactions, in cellular environments it may be more likely to be present as some kind of bidentate ligand and catalysing other reactive species, such as alkoxyl (RO) or peroxyl radicals (ROO) that could also occur during the porphyrogenic process [196]. Following HCB-induced porphyria in rats, iron and porphyrins accumulate in hepatic lysosomes indicating intracellular mobilisation and transport [221]. Other indicators of disturbance of iron metabolism associated with sPCT [46] and experimental porphyria are supported by changes in binding of iron regulatory proteins and changes in expression of hepcidin [222,223,224].

Understanding the participation of iron in metabolic dysfunction has greatly evolved in recent years. This is not a review of ferrotopsis, but it would seem likely that there are many aspects of the mobilisation and role of toxic iron in the development of uroporphyria, which are in common with this route of cell death [225,226,227]. Repercussions of iron-catalysed oxidation of unsaturated fatty acids in lipids are well studied features of ferritinophagy and ferroptosis. In comparison, oxidation of the sensitive porphyrinogens (uroporphyrinogen III, coproporphyrinogen III and protoporphyrinogen IX) intermediates of heme synthesis are not considered, although they are stages in a critical pathway for cellular metabolism.

Nutrition is well known to influence markedly acute hepatic porphyrias and indeed control of some dietary constituents can help considerably in managing AIP [5,228]. Some of this is due to regulation of heme biosynthesis via peroxisome proliferator-activated receptor gamma coactivator 1alpha (PGC-1alpha) [229]. Besides the pathogenic accumulation of iron exhibited in some sPCT patients carrying the *HFE* Cys282Tyr variant, there could well be other nutritional factors that have yet to be investigated and compatible with the acquired nature of the porphyria, perhaps influencing the mobilisation of redox active iron. Recently, the response of C57BL/10ScSn mice to iron overload causing uroporphyria was reported to differ between animals fed a basic common maintenance laboratory diet and the same diet with added nutritional value to enhance breeding and growth [230]. Uroporphyrin accumulated in the liver of those mice fed the enhanced diet implying that some aspect of metabolism, perhaps metabolic rate, accelerated iron-catalysed uroporphyrinogen oxidation. If control mice received drinking water containing 5-ALA to accelerate heme synthesis, protoporphyrin accumulated with either diet suggesting that iron availability was limiting the formation of heme [231]. In contrast, if those mice fed the basal diet were given iron little protoporphyrin was observed in the liver but uroporphyrin occurred with the enhanced diet. Total iron levels were not different. However, the isomers of uroporphyrin formed were not determined in these experiments and it might prove important to understand their origin whether as consequences from depletion of UROS or UROD activities. Interestingly, chronic administration of 5-ALA to C57BL/6N reportedly enhances hepatic cytochrome c oxidase activity and ATP levels illustrating the possibility that the heme precursor has other mechanisms of action [232].

Although the involvement of iron is assumed to be of a labile catalytic free radical nature, the complexity of intracellular hepatic iron homeostasis could mean that its influence in sPCT is more nuanced, perhaps in the regulation of mitochondrial activity leaking ROS [10]. Iron from the action of heme oxygenase 1 could be involved in the process of uroporphyrinogen oxidation. Current knowledge of HMOX1 demonstrates its complex role in oxidative stress phenomena [233]. Although not definitive, findings with uroporphyra development in experimental models have proven inconsistent [206,230,234]. In the development of new mammalian in vitro systems to study the role of iron in the oxidation of uroporphyrinogens, the decreased activity of UROD could prove beneficial, perhaps employing hepatic organoids of defined genetic and lobular origin [235,236,237].

## 6. Genetic Susceptibility

Uroporphyrinogens are sensitive to oxidation but this does not usually seem to be a problem although there appears to be a variable predisposition, particularly in the liver with high heme synthesis for cytochromes. In sPCT, a wide variety of exposures or conditions, including, alcohol, estrogenic drugs, HIV, hepatitis C and dialysis that may affect iron metabolism, are implicated, but stimulation of oxidation only occurs in occasional individuals and under certain physiological circumstances [46]. Carrying the *HFE* mutations that are associated with massive elevations of hepatic iron is foremost of those inherited factors in patients with European ancestry but increased iron levels themselves may not be enough [16,46,47,49,53,222]. Mutation studies of promoter regions of some other genes involved in iron homeostasis, ceruloplasmin, cytochrome b reductase 1, hepcidin, and solute carrier family 40A1, found some association with PCT in a mixture of indigenous, white and mixed races patients [52,222,238]. USA PCT patients of both familial (fPCT) and acquired type (sPCT) were associated with a CYP1A2 promoter variant and wild type glutathione S-transferase Mu1 [239]. Polymorphism of a CYP1A2 intron was increased in a collection of Danish patients but CYP1A2 genotype variants did not associate strongly with either fPCT or sPCT in those from France and Spain [240,241,242]. CYP1A2 variants and activities are highly variable in human populations for not altogether understood reasons and are not just dependent on the AHR [243]. As noted earlier, a pathogenic UROD variant itself can of course be a genetic contribution to overt PCT [244]. On the other hand, although widespread, the incidence of porphyria in Türkiye was not directly dependent on the consumption of HCB-contaminated wheat by the exposed population but seemed to depend on family, sex and age factors. Most of those affected were children in contrast to PCT in which patients are mostly older adults [14,245]. Although still very important, this wide-spread episode of toxic porphyria occurred before modern methods of data collection and genetic analysis. There are many aspects yet to be explained as to why HCB is so active in this respect in humans, rats and mice, but not hamsters, depending on sex or strain, that does not seem accountable by just expression of CYP1A2.

Although moderate iron overload is associated with both forms of human PCT, and iron enhances the effects of experimental uroporphyria, it is clear that iron levels per se are not predictive and other genetic loci besides those involved in iron accumulation must be involved [222]. Alcohol consumption is a major factor in many cases of sPCT but few studies in rodents have shown it to be uroporphyric even with iron overload [246]. Eventually, a uroporphyric response to alcohol in *Hfe* knockout mice was shown but was dependent on the genetic background of the null strain. Further investigation of this model should prove useful to understand the nature and site of the iron involved [247].

Sweeney and colleagues showed that in crosses between sensitive C57BL/6J and resistant DBA/2 strains susceptibility to the uroporphyria in F2 mice caused by TCDD was inherited associated with the responsiveness to induction of CYP1A1 activity [95,248]. Classically, C57BL/6J mice are responsive and DBA/2 nonresponsive determined by polymorphisms of the aryl hydrocarbon receptor gene (*Ahr*). The difference of inbred mouse strains to respond to TCDD induction of CYP1A1 activity depends on the binding affinities of the different AHR proteins coded by polymorphic alleles (*Ahr b-1*, *b-2* and *d*) [249,250]. Mice like C57BL/6J with high affinity AHR *b-1* are responsive whereas those with low affinity AHR *d*, such as DBA/2, are nonresponsive, differing by a factor of up to 20. At higher doses maximum induction is nearly the same. On the other hand, the difference in susceptibility to uroporphyria between the related *Ahr b-1* strain C57BL/10ScSn and DBA/2 mice is >20-fold [90]. In fact, when comparing a wide range of iron-treated inbred strains, susceptibility to TCDD- and HCB-induced porphyria does not correlate with the AHR phenotype or *Ahr* genotype although DBA/2 is the most resistant [96,100,179,224,251]. In particular, the susceptibility of another *Ahr d* strain SWR in the presence of iron, compared to DBA/2, confirmed that there must be genes other than those governed by the AHR and the inducibility of CYP1A activity to make the latter strain highly resistant. Knockout of the *Ahr* gene in C57BL/6J mice has a marked effect on TCDD porphyria but not totally on other stimuli [234]. Comparison of TCDD-induced porphyria in C57BL/6J with DBA/2 mice, showed that there are many differences in up- and downregulation of gene expression together with decreased activity of glutathione peroxidase activity and aconitase (iron regulatory protein IRP), as occurs in ferrotoptosis [224,234]. TCDD also causes a decline of IRP/IR element interaction as well as total IRP capacity in C57BL/6J mice but significantly less in DBA/2. It has not been resolved whether this is a mechanism or consequence of porphyria.

That there is a genetically variable hepatic response to just iron in mice independent of *Ahr* genotype was confirmed by the eventual UROD inhibition and uroporphyria after iron overload alone in C57BL/10ScSn and SWR mice but not in DBA/2 [99,105,106,107]. It is an important mechanistic point that oral co-administration of 5-ALA not only speeds up this response but that with the heme precursor on its own uroporphyrinogens are oxidised to uroporphyrins (and UROD is inhibited) in SWR mice whereas protoporphyrin IX accumulates in DBA/2 liver [110]. Understanding this difference would be an important clue to the whole process of uroporphyria development. What might be relevant to the resistance of the DBA/2 strain is the low rate of microsomal lipid peroxidation, NADPH oxidation and oxygen consumption in control and after iron compared with *Ahr b-1* C57BL strains and AKR mice (*Ahr d*) [175,252,253]. Another potential aspect would be differential clearance of coproporphyrinogen into the mitochondria due to variants of the gene for ABCB6 [254].

Quantitative trait loci analysis (QTL) of induction of uroporphyria caused by HCB, TCDD and even just 5-ALA, in F2 crosses from iron loaded susceptible strains with the resistant DBA/2 strain has confirmed that susceptibilities are associated with multiple genetic loci [121,230,255,256]. Some appear in common but others vary with strain and treatment. Only with HCB and TCDD and in C57BL strains does polymorphism of the *Ahr* gene play a significant role in susceptibility. In addition, from these QTL crosses there is poor evidence that differential protein, activity or gene expression of CYP1A2 explains the resistance of the DBA/2 strain. After uroporphyria induced by iron/5-ALA, transcriptomics of parents and F2 QTL confidence regions showed differential expressions of genes on chromosomes 1, 11, and 17 that might account for susceptibility of SWR mice relative to DBA/2. Some of these are associated with oxidative responses or mobilisation of iron, transport and glutathione metabolism [230]. One observation was marked decreased expression of *Glul* for perivenous (zone III) glutamine synthetase and was also a feature of uroporphyria in C57BL/10ScSn with iron overload when maintained on an enhanced diet [230,257]. QTL may not only demonstrate polymorphisms associated with differences in gene expression but others that are not detectable (as with *Ahr*), or noncoding regulatory sequences. More detailed summary of QTL and discussion associated with uroporphyria are described elsewhere [230,255,256].

Most of the experimental studies of the formation of uroporphyrinogens have focussed on the genetic susceptibility mechanisms of oxidation. However, how the liver cell protects HMB and uroporphyrinogens from oxidation during heme synthesis is an important issue but has not been studied methodically and with tools, such as mice with compromised antioxidant systems [258,259]. Under circumstances in which heme synthesis is stimulated, as in the demand for mitochondrial and microsomal cytochromes, the levels of porphyrinogens formed may be much greater than UROD can process and overwhelms protection mechanisms.

## 7. Consequences

The oxidation of uroporphyrinogens during heme synthesis is usually minor but under pathological conditions the process or the resulting uroporphyrins and other products have the potential to be detrimental to cellular health, especially in the liver [14,46,101,141,224,260]. This is aside of phototoxic injury seen in CEP, PCT and Turkish HCB porphyria due to the cutaneous deposition of uroporphyrinogens and uroporphyrins [8,15]. Mild to moderate aspects of liver damage are reported in sPCT patients but it is difficult to distinguish these from the effects of the triggering agents [15,17,246,261,262,263]. In both experimental porphyria and sPCT, uroporphyrin crystals are observed in hepatocytes, often associated with ferritin-like deposits of iron, suggesting a possible mechanistic link [106,264,265,266,267]. Uroporphyria induced in rodents by HCB and TCDD modulated by iron is associated with markers of liver injury but are not completely interdependent [96,98,175,256].

sPCT patients also have a raised risk of liver cancer [268]. In rats the marked sex difference in chemical-induced uroporphyria is also seen in the incidence of liver tumors [85,260]. The potentiation by iron overload of the HCB- or PCBs-induced porphyria in C57BL/10ScSn mice is reflected in a marked increase in the numbers and severity of hepatocellular tumors whereas DBA/2 and CYP1A2 knockout mice are resistant to both [18,99,101,139]. Whether uroporphyria is related directly to the reported higher risk of liver cancer in PCT patients requires further long-term studies and understanding of the mechanism in rodents [260,268]. If uroporphyrins themselves cause these pathological findings, how does this happen at a molecular level? Uroporphyrins are much more hydrophilic than protoporphyrin and less likely to cause liver injury by precipitation. If the mechanism is the consequence of a common or concomitant underlying iron-catalysed oxidative process, which can cause porphyria and liver tumours, it would have other fundamental implications. Both phenomena are important to understanding the true hepatic cancer risks for humans from exposure to chemicals, such as HCB, TCDD and drugs that also cause uroporphyria. Understanding the relevance of molecular modes of action for rodent liver tumours caused by chemicals is an important aspect of human risk assessment but plausible mechanisms are easier to propose than prove [109].

## 8. Conclusions

**Figure 6:**
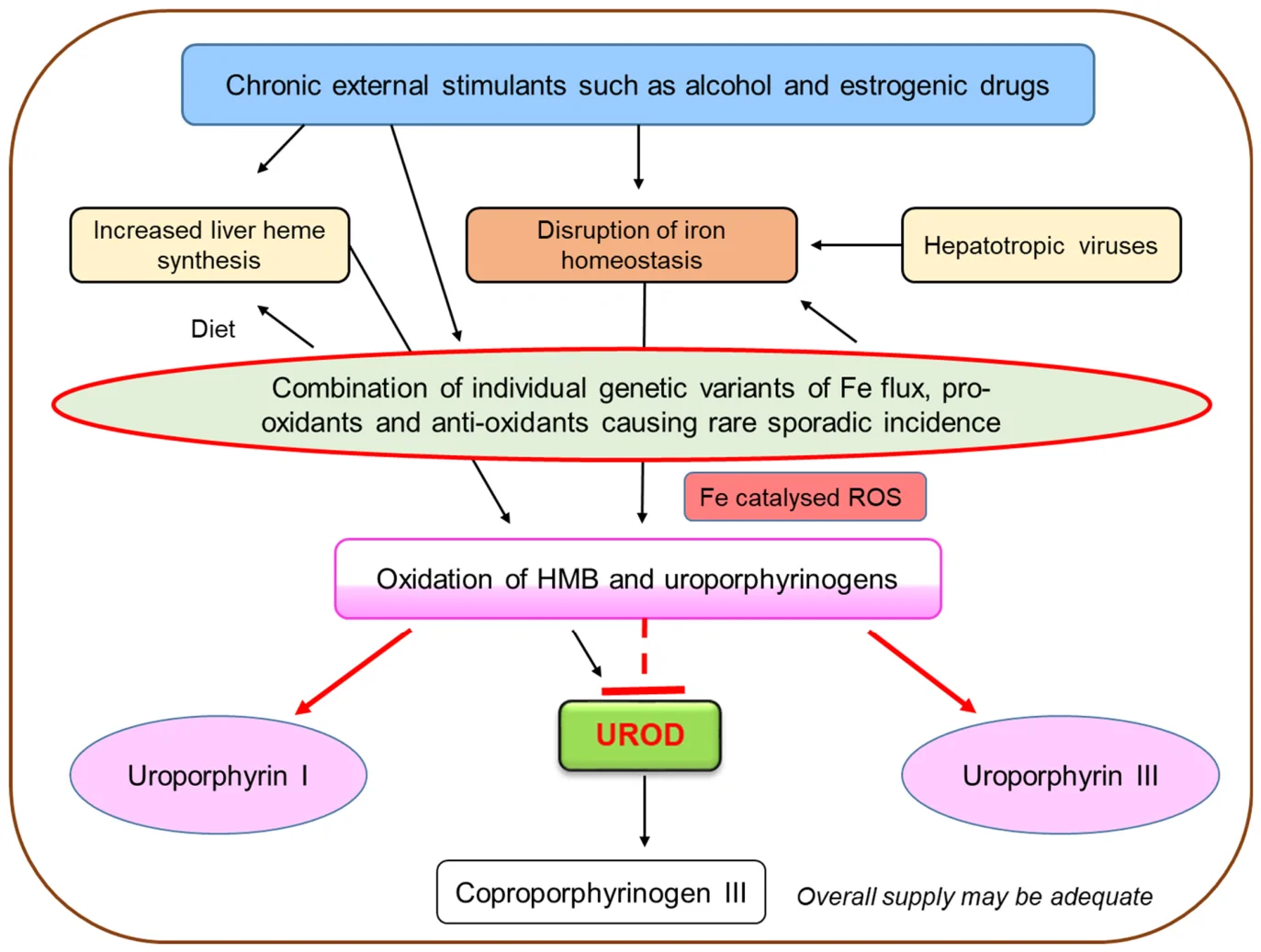
The incidence of sPCT is sporadic and rare, probably due to a combination of variable genetic and physiological factors that at the present time are not predictable. Contributing factors can include iron overload as a consequence of *HFE* mutations, and alcohol consumption. The development of uroporphyria and UROD inhibition in experimental animals is much more predictable due to the selection of susceptible species, sex and strain, and porphyric regimens, especially including powerful chemical inducers, such as HCB.

Uroporphyrinogen III formed enzymatically from HMB as a cytosolic intermediate in heme synthesis, is prone to oxidation to the respective uroporphyrin III via partly oxidised intermediates. HMB can also be cyclised nonenzymatically to the symmetrical isomer uroporphyrinogen I, which is not further utilised. Minor oxidation products of uroporphyrinogens include oxy- and hydroxy nonporphyrin products. Trace amounts of uroporphyrins and coproporphyrins are excreted normally. In a very few people insufficient activity of hepatic uroporphyrinogen decarboxylase (UROD) is associated with a massive accumulation of uroporphyrinogens and uroporphyrins in liver and skin and high urinary excretion, particularly in the cutaneous porphyria PCT (Figure 6).

The majority of patients are idiopathic (sPCT) and do not carry a pathogenic *UROD* variant. HCB and related chemicals cause a similar disorder in people and rodents. The issues of the susceptibility and iron factors accounting for why sPCT is sporadic have been debated for decades [46]. In experimental studies, low UROD activity may be due to what has been reported as a uroporphomethene inhibitor, a partly oxidised uroporphyrinogen. Many in vitro and in vivo investigations have focussed on the role of CYP1A2 in the oxidation of uroporphyrinogens. However, there is still uncertainty around the role of CYP1A2 in rodent porphyria and whether this is relevant to humans. Iron metabolism is implicated in both sPCT and in chemical-induced uroporphyria in rodents. Under chronic siderotic conditions in certain mice, alcohol, and some other drugs and nonchlorinated chemicals are porphyrogenic. Iron alone overload eventually causes this response in susceptible mice implying that iron metabolism may underpin the mechanism of action of the large variety of triggering agents, such as alcohol, estrogens, chemicals and hepatotrophic viruses. The use of the term ‘sporadic’ PCT implies that the frequency is unpredictable and rare requiring the combination of external and unknown genetic predisposing factors (Figure 6). This resembles rare off-target responses to drugs in patients. The combination of a number of susceptibility loci in iron-dosed mice, including *Ahr*, accounts for the difference in susceptibility between some strains but also depends on nongenetic triggers. These loci may represent differential expression of pro- or antioxidant genes, including mobilisation of iron from unknown sites. Their identification would contribute important information to comprehend the complete mechanism of experimental porphyria and the sporadic nature of sPCT. Understanding the porphyria following exposure of people and rodents to HCB and related chemicals is an important aspect of determining their mode of action and risk assessment.

## Figures and Tables

**Table 1:** Major mammalian in vivo circumstances for the deposition and urinary excretion of uroporphyrins derived from oxidation of uroporphyrinogens.

Disorder	Gene Component	Enzyme and Main Site Affected	Main Uroporphyrin and Products Detected In Vivo *
Congenital eryrthropoietic porphyria (CEP)	*UROS*	Uroporphyrinogen III synthase variant. Bone marrow (AR)	Uroporphyrin I >> III + partial decarboxylated I
Hepatoerythropoietic porphyria	*UROD*	Uroporphyrinogen decarboxylase variant. Liver (AR)	Uroporphyrins I III + partial decarboxylated
Familial porphyria cutanea tarda	*UROD*	Uroporphyrinogen decarboxylase variant. Liver (AD)	Uroporphyrins I III + partial decarboxylated
Sporadic (acquired) porphyria cutanea tarda	-	Uroporphyrinogen decarboxylase inhibited. Liver	Uroporphyrins I III + partial decarboxylated *
Toxic human porphyrias (HCB, TCDD, PCBs)	-	Presumed inhibited uroporphyrinogen decarboxylase. Liver	Uroporphyrins I III **
Rodent porphyria cause by HCB, TCDD, PCBs	-	Uroporphyrinogen decarboxylase inhibited. Liver	Uroporphyrins I III + partial decarboxylated

* Derived from oxidation of the uroporphyrinogens. In CEP, uroporphyrinogen I is formed by the spontaneous cyclisation of the excess hydroxymethylbilane arising as a consequence of the UROS III variant with low activity. ** No detailed examination of isomers and partial decarboxylation products. As well as urinary excretion, deposition in skin and elsewhere leads to photosensitivity. AR, autosomal recessive. AD, autosomal dominant.

## Data Availability

No new data were created or analysed in this study.

## Abbreviations

The following abbreviations are used in this manuscript:

5-ALA	5-Aminolevulinic acid
5-ALAS	5-Aminolevulinic acid synthase
AHR	Aryl hydrocarbon receptor
CEP	Congenital erythropoietic porphyria
HCB	Hexachlorobenzene
HMB	Hydroxymethylbilane
HMBS	Hydroxymethylbilane synthase
PCBs	Polychlorinated biphenyls
PCT	Porphyria cutanea tarda
PPOX	Protoporphyrinogen oxidase
ROS	Reactive oxygen species
QTL	Quantitative trait loci
TCDD	2,3,7,8,-Tetrachlorodibenzo-*p*-dioxin
UROD	Uroporphyrinogen decarboxylase
UROS	Uroporphyrinogen synthase
